# Analysis of 5’ gene regions reveals extraordinary conservation of novel non-coding sequences in a wide range of animals

**DOI:** 10.1186/s12862-015-0499-6

**Published:** 2015-10-19

**Authors:** Nathaniel J. Davies, Peter Krusche, Eran Tauber, Sascha Ott

**Affiliations:** Department of Genetics, University of Leicester, Leicester, UK; Warwick Systems Biology Centre, University of Warwick, Coventry, UK

**Keywords:** Conserved non-coding regions, *Nasonia vitripennis*, Phylogenetic footprinting, Translation regulation, mRNA secondary structures, UTRs

## Abstract

**Background:**

Phylogenetic footprinting is a comparative method based on the principle that functional sequence elements will acquire fewer mutations over time than non-functional sequences. Successful comparisons of distantly related species will thus yield highly important sequence elements likely to serve fundamental biological roles. RNA regulatory elements are less well understood than those in DNA. In this study we use the emerging model organism *Nasonia vitripennis*, a parasitic wasp, in a comparative analysis against 12 insect genomes to identify deeply conserved non-coding elements (CNEs) conserved in large groups of insects, with a focus on 5’ UTRs and promoter sequences.

**Results:**

We report the identification of 322 CNEs conserved across a broad range of insect orders. The identified regions are associated with regulatory and developmental genes, and contain short footprints revealing aspects of their likely function in translational regulation. The most ancient regions identified in our analysis were all found to overlap transcribed regions of genes, reflecting stronger conservation of translational regulatory elements than transcriptional elements. Further expanding sequence analyses to non-insect species we also report the discovery of, to our knowledge, the two oldest and most ubiquitous CNE’s yet described in the animal kingdom (700 MYA). These ancient conserved non-coding elements are associated with the two ribosomal stalk genes, *RPLP1* and *RPLP2*, and were very likely functional in some of the earliest animals.

**Conclusions:**

We report the identification of the most deeply conserved CNE’s found to date, and several other deeply conserved elements which are without exception, part of 5’ untranslated regions of transcripts, and occur in a number of key translational regulatory genes, highlighting translational regulation of translational regulators as a conserved feature of insect genomes.

**Electronic supplementary material:**

The online version of this article (doi:10.1186/s12862-015-0499-6) contains supplementary material, which is available to authorized users.

## Background

The orchestration of gene expression is accomplished through a wide variety of regulatory mechanisms. One of the most well-characterized of these mechanisms is regulation of transcription through the binding of transcription factors to regulatory DNA sequence [1]. The DNA sequences bound by transcription factors are generally short, the average binding site length being around 10 bp in eukaryotes [2]. Other types of regulatory sequences are less well characterized; for example those sequences which become functional when transcribed into RNA. The most well-known example of this kind of regulatory element is perhaps the IRE (iron response element), a hairpin loop found in the mRNA of many genes involved in iron metabolism which helps to maintain iron homeostasis [3].

Detecting regulatory elements experimentally is time consuming [4], and identifying appropriate experimental targets may be difficult. Using straightforward computational methods for prediction of regulatory elements also presents issues; for example, prediction of transcription factor binding sites is usually accomplished by scanning a sequence of interest for matches to position-specific scoring matrices (PSSMs). These PSSMs [5] describe the kinds of, generally short [2] sequence motifs bound by these proteins. As such, the probability of finding a chance match in a sequence of any considerable length is high, and the majority of predicted transcription factor binding sites are therefore likely to have no functional role; a concept dubbed the ‘futility theorem’ [6]. Many other regulatory elements are also characterized by short sequence motifs, and so identification of these elements through straightforward sequence scanning methods is subject to the same problem.

Phylogenetic footprinting is a method that can greatly reduce the search space when looking for functional regulatory elements [7]. It is based on the principle that functionally important sequence elements are more likely to be conserved over time than less (or non-) functional elements, leaving behind a ‘footprint’ of functionality. This approach can be highly successful at identifying functional regulatory elements, the specificity of detection increasing with the divergence times of the species used; for example >40 % of conserved non-coding elements (CNEs) detected through a human-fugu (454.6 Myr divergence) [8] comparison showed enhancer activity when tested [7], as opposed to only 5 % of human-rodent CNEs tested [9]. CNE detection tends to drop sharply at taxonomic boundaries, for example sensitive BLAST analysis shows a clear alignment signal between similar loci of two Drosophila species (~60 Myr), but an almost complete lack of alignment between two more diverged dipteran species (~75 Myr) [10].

The most deeply conserved CNEs detected to date originated before the divergence of deuterostomes and protostomes, only four examples of which have been found so far. The first two sequences of this kind to be discovered were found conserved between a variety of deuterostomes and a cnidarian, *Nematostella vectensis* [11], dating back over 670 million years [12]. The other two conserved sequences that predate this split were, unlike the other two sequences, found to be present in species belonging to both Deuterostomia and Protostomia [13] and date back at least 600 million years [12].

Here, we took advantage of the recent releases of various insect genomes to identify novel regulatory elements conserved across large (180–700 myr) evolutionary distances. The majority of phylogenetic footprinting studies in insects use the model organism *Drosophila melanogaster* as a central comparison species, aimed at finding regulatory elements conserved within the fast-evolving [14] order Diptera. For a new perspective, we here use the emerging model organism *Nasonia vitripennis*, a member of the more slowly evolving order Hymenoptera, as a central comparison species to identify conserved regulatory elements. The aim of this study was to characterize a small subset of deeply conserved sequences in the upstream region of genes, thus potentially capturing both novel transcriptional and translational regulatory elements. By using a sensitive alignment algorithm (see Methods) [15] and ensuring that our analysis was conducted with a low false discovery rate, we identified a set of conserved sequences. Among the sequences that we identified are both known regulatory elements and a variety of novel regulatory elements on or near genes with core regulatory or developmental roles, some of which could potentially represent novel classes of RNA regulatory elements. We use our set of CNEs to examine the nature of conserved regulatory elements and their evolution. We also report the discovery of, to our knowledge, the two most deeply and ubiquitously conserved regulatory elements yet identified in the animal kingdom which date back to the radiations of basal animal phyla and are likely over 670 [12] and 700 million years old [8] respectively.

## Results

### Identification of deeply conserved non-coding elements

**Fig. 1:**
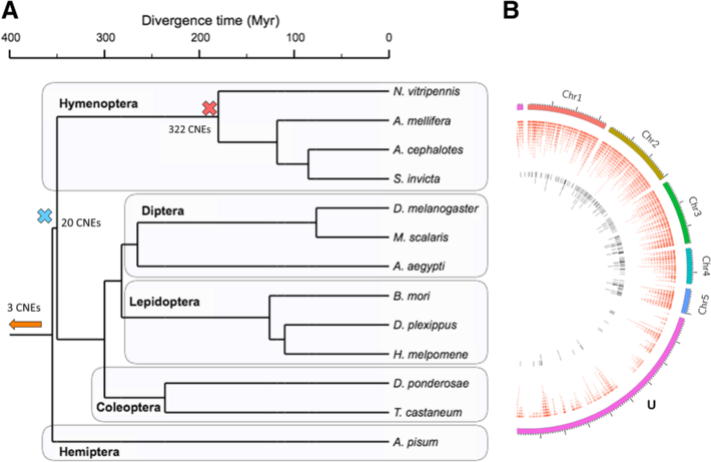
Phylogenetic relationships between species and genomic CNE distribution. **a** Phylogenetic tree showing the relationships and approximate divergence times between the insects used in the analysis. The number of conserved CNEs at different branching points is plotted on the figure. Phylogenetic relationships and divergence times from: inter-order [8]; Hymenoptera [16]; Lepidoptera [57]; Diptera [58]; Coleoptera [8]. **b** *Nasonia vitripennis* genome diagram showing the locations of the sequences analyzed (red, outer circle, 3.66 % of genome) in contrast with the sequences identified as conserved (black, inner circle, 0.0064 % of genome). Lower CNE density on chromosome U reflects lower gene density on these unplaced scaffolds

In order to identify conserved regulatory elements, we performed a comparative analysis of 13 highly diverged insect genomes (Fig. 1a) on a locus-by-locus basis, scanning the 2 kb non-coding region upstream of the translation start site of each gene. *N. vitripennis* was used as the central species compared to all other species in a series of pairwise comparisons. The “seaweed algorithm" [15] was used to perform alignments, performing over 3.8 million optimal alignments of short sub-sequences per pair of 2 kb sequences upstream of orthologous genes. Significantly aligned, overlapping sub-sequences were merged and regions in other species that mapped to the same sub-sequence in *N. vitripennis* were identified to yield one inclusive dataset (see Methods).

As a control, we aligned pairs of randomly matched upstream non-coding sequences. The number of ‘conserved’ sequences detected in the control set at various alignment score thresholds can therefore be used to estimate the false discovery rate. We adjusted the algorithm parameters such that no conservation at all was detected in the control, and then used these parameters to align the truly orthologous sequences. Sequences were pre-filtered for repetitive regions, and post-filtered for similarities to known coding sequences. At this very strict level of false discovery, we detected 322 CNEs on or near 276 genes (Fig. 1b). Each of these genes was given a *combined conservation score* (CCS) in the interval 0–1, where anything above zero is considered statistically significant and one represents particularly strong conservation (see Methods).

Since the most closely related species in the analysis (the three Hymenopteran species) diverged from Nasonia approximately 180 million years ago [16], all of the 322 CNEs have been conserved for at least this long. The CNEs found in Hymenoptera tend to be found in more than just two species; ~58 % of the hymenoptera-specific CNEs are conserved in three species or more (*N. vitripennis* and two others). Focusing on the 276 genes with an associated CNE, we expanded the analysis to a wider range of animals. A handful of CNEs were found to be conserved at greater evolutionary distances; 20 CNEs on or near 18 genes were found to have been conserved for at least 350 million years (i.e. the common ancestor of Holometabola) [17]. Of these, one CNE dates back to the common ancestor of Mandibulata (myriapods, crustaceans, and hexapods), and 2 CNEs date further back to the radiations of basal animal phyla (Cnidaria, Placozoa, Ctenophora, and Porifera). These 20 anciently conserved CNEs exhibit a high degree of overlap, with only two being specific to *N. vitripennis* and one other species.

### Relative position of CNEs is conserved along with sequence

In order to investigate the properties of the CNEs, we performed a series of analyses comparing the CNEs with a control set of sequences. To obtain these control sequences, we adjusted the parameters of the algorithm to allow for the capture of false discoveries, and ran the alignments on randomly matched (pseudo-orthologous) pairs of sequences. By setting an appropriate threshold, we extracted and post-filtered a similar number of sequences to the CNEs from the control set, representing the highest scoring non-orthologous sequence alignments. We term these sequences *pseudo-CNEs*, as they are sequences that have high alignment scores, albeit below our conservation threshold, but lack orthology. As high-scoring non-orthologous sequences, these pseudo-CNEs can be used as a comparison to elucidate important sequence properties about the true, orthologous CNEs, as opposed to comparisons with sequences with randomized properties.

**Fig. 2:**
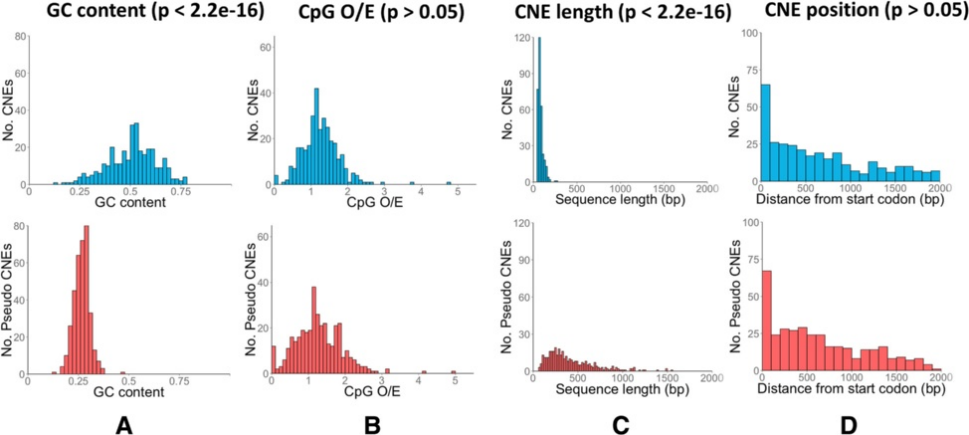
Comparative analysis of CNE sequence features. Analysis of CNE (blue) properties in comparison with pseudo-CNE controls (red). Distributions were compared using the Wilcoxon rank-sum test. **a **GC content. **b** CpG O/E. **c **CNE length distribution. **d** CNE position

The GC content of the CNEs contrasts starkly with the GC content of the pseudo CNEs; the CNEs have a mean GC content of 51 %, compared to 27 % in the pseudo CNEs (Fig. 2a). This pattern of GC content is strongly associated with a peak of predicted nucleosome occupancy in the center of the CNE (Additional file 1: Figure S1), a markedly different population of over/under-represented transcription factor binding sites (Additional file 1: Figure S2), and an underrepresentation of ATG trinucleotides (Additional file 1: Figure S3), although whether these are a cause or effect of the GC content disparity is unclear. The expected number of CpG dinucleotides based on the GC content (CpG O/E) in the CNEs does not significantly differ from the control (Fig. 2b), suggesting that there is no suppression or special use of these methylation-related dinucleotides in the conserved regions. The length of the CNEs is strongly biased towards ~90 bp, a trend which is not generally apparent in the pseudo-CNEs (Fig. 2c), perhaps indicative of the mode of mechanism of these conserved sequences.

**Fig. 3:**
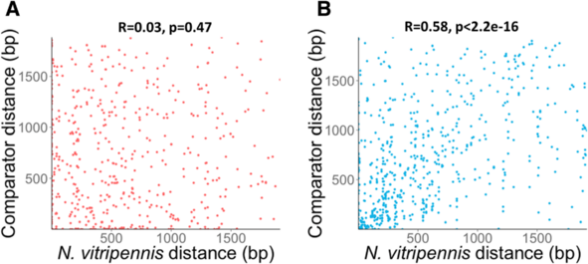
Conservation of relative CNE positions. Scatter plot showing the conservation of CNE positions, comparing the CNE position in *Nasonia* (x-axis) with its position in each comparator organism (y-axis). Conservation of position relative to translation start site is insignificant in the control (**a**, *p* = 0.47) but significant among CNEs (**b**, *p* < 2.2e-16)

When we consider the distance of the sequences from the translation start site of their associated gene, we see enrichment for adjacency among both the CNEs and the pseudo-CNEs, decreasing with distance (Fig. 2d). Although the relative positions of the CNEs and the pseudo-CNEs are similar, the conservation of these distances across species is not. Comparing the translation start site distance in *N. vitripennis* with the translation start site distance in the comparator species reveals that the distance of each pseudo-CNE in *N. vitripennis* is completely uncorrelated with its distance in the comparator organism (Fig. 3a), whereas in the set of CNEs there is a significant correlation between distances (Fig. 3b). This result shows that the position of CNEs is important as well as the conserved sequence itself.

### CNEs are tightly associated with developmental and regulatory genes

The 322 CNEs that we identified here are associated with a specific class of genes. We tested for overrepresentation of gene ontology (GO) terms against the genomic background using the annotation information available (see Methods) for each *N. vitripennis* gene associated with a conserved region. 319 terms were significantly overrepresented with a q-value below 0.01. The most overrepresented term in the set (Additional file 2: Table S1) was ‘regulation of gene expression’ (q < 2.7e-31) which was associated with over a third (36.7 %) of the genes tested. In addition, many significant terms such as ‘nucleic acid binding transcription factor activity’ (q < 3.7e-28, 21.9 % of genes tested) and ‘developmental process’ (q < 8.8e-30, 51.5 % of genes tested) were returned, suggesting that genes associated with upstream conserved regions often themselves have regulatory and/or developmental roles. A set of 28 terms were overrepresented for the set of 359 pseudo-CNEs (Additional file 2: Table S2), albeit with lower significance compared to the CNE set. This suggests that the long highly AT-rich sequences that are picked up in this control have a weak, but detectable association with gene expression and specific processes – an observation not further explored here.

The 20 most deeply conserved sequences (> = 350 Myr) also appear to be associated with a specific class of genes. 14 of these CNEs were found to lie completely within transcribed regions (see Methods), and all 20 were found to overlap transcribed regions by at least a third of the length of the CNE. This enrichment is significant (*p* < 6.5e-04, hypergeometric test) when compared to the full set of 322, of which only ~70 % overlap transcribed regions by this amount. Remarkably for such a small set of genes, a GO term overrepresentation test turned up 39 significant terms. The 17 genes associated with these 20 CNEs are enriched for genes active in processes such as post-transcriptional regulation of gene expression (q < 6.1e-4), regulation of translation (q < 4.8e-3), and translational elongation (q < 1.2e-2). This list of overrepresented GO terms is, unlike that obtained from the full set of 322 CNEs, devoid of terms relating to transcriptional regulation, matching the shift towards putative translational regulatory CNEs.

### 5’ UTR CNEs contain conserved secondary structures

**Fig. 4:**
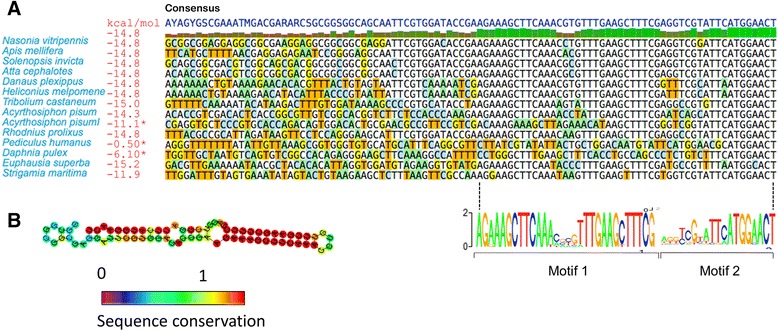
A highly conserved hairpin loop in the 5’ UTR of Not1. **a** Upstream CNE-containing Not1 sequences. Three footprints of conservation are clearly visible; the first two (from left) constitute the stem sequence of the hairpin and are shown as motif 1. The third footprint (shown as motif 2) is the conserved sequence adjacent to the translation start site and contains an ATG upstream of the translation start site. Hairpin loop stabilities are shown in red and outliers (disrupted loops) are marked with blue asterisks. Acyrthosiphon pisumI represents a putative Not1 paralog. **b** N. vitripennis Not1 CNE predicted RNA folded structure colored by sequence conservation, showing highly variable apical sequence and conserved stem

Among the CNEs that we identified were previously-studied regulatory elements, as well as many unidentified novel putative regulatory elements. As the majority of CNEs overlap 5’ UTRs, we calculated the likelihood of there being a conserved secondary structure in each CNE. This analysis revealed several conserved secondary structures, including an example of the well-characterized iron response element (IRE) in the 5’ UTR of the *Ferritin* gene (Additional file 1: Figure S4), a conserved hairpin loop bound by iron response proteins (IRPs) to help maintain iron homeostasis. We also identified novel conserved RNA structures, including a conserved, strong (−52.60 kcal/mol) hairpin loop found in the 5’ UTR of the *Paramyosin* gene (Additional file 1: Figure S5) identified in all four Hymenoptera species, and a hairpin loop with perfect stem complementarity but variable apical sequence conserved in the 5’ UTR of the *Not1* gene (Fig. 4). These three hairpins differ in their fundamental characteristics. IREs are characterized by a highly conserved apical sequence (CAGUGY; clearly demonstrated in the three hymenopteran species) with a more variable stem sequence [3]. In contrast, the 4-nucleotide apical sequence (consensus HVHN) of *Not1* appears to be highly variable, whereas the stem sequence is almost perfectly conserved. More sequences are necessary to be able to reliably characterize the *Paramyosin* hairpin, although there does appear to be at least one variable nucleotide in the hairpin apex. The positions of the hairpins also appear to be of functional importance; all three hairpins are conserved in their position relative to the translation start site, particularly the *Not1* hairpin (Fig. 4a).

The *Not1* hairpin loop has a stem sequence of 12 bp, and the CNE containing it is found directly adjacent to the translation start site. The CNE contains two conserved stem sequences with near-perfect complementarity, a weakly conserved apical sequence, and a highly conserved, upstream ATG-containing motif directly adjacent to the translation start site. In *N. vitripennis*, this CNE is present in the 5’ UTR of all four known transcripts. As the position of this CNE is so strongly conserved, we scanned the first 100 bp of every orthologous transcript in all Ensembl Metazoa species for presence of either the conserved hairpin or for the conserved sequence adjacent to the translation start site. The results of this search (see Methods) indicated that in all cases where the hairpin loop is present the conserved sequence adjacent to the translation start site is present too, but not vice-versa (i.e. the sequence adjacent to the translation start site may exist on its own). The presence of the sequence in the Antarctic krill *Euphasia superba* (Hunt and Rosato, unpublished data) and in a centipede (*Strigamia maritima*) shows that this CNE was an early arthropod adaptation.

### An uncharacterized gene cluster contains several CNEs

We identified conserved putative regulatory sequences in six separate genes of the insect-specific *Osiris* gene cluster (Additional file 1: Figure S6). Our analysis indicates that these regions are Hymenoptera-specific, and are generally conserved in position relative to their associated gene. Since the conserved regions are associated with a specific class of genes with core functions, the fact that conserved promoter regions were identified near to six genes in the same cluster is perhaps indicative of an important developmental or regulatory role for this as-yet uncharacterized gene cluster.

### Ribosomal stalk gene CNEs date back to early animals

**Fig. 5:**
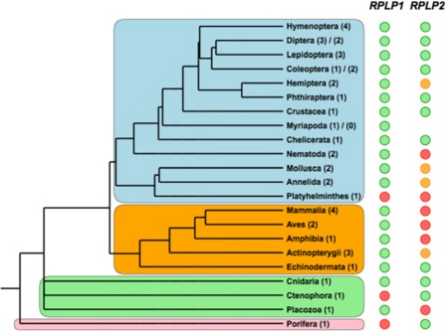
Distribution of most ancient CNEs across phyla. Cladogram showing presence (green), absence (red), or mixed presence-absence (orange) of the deeply conserved RPLP1 and RPLP2 CNEs. Numbers in brackets show number of species analyzed per group for the RPLP1/RPLP2 CNEs respectively. Groups are outlined by color: blue (Protostomia), orange (Deuterostomia), green (Cnidaria, Ctenophora, and Placozoa), pink (Porifera)

Two conserved sequences were identified in the 5’ UTRs of the two ribosomal stalk heterodimer genes, *RPLP1* and *RPLP2*. Given that parts of these sequences were found to be perfectly conserved over several nucleotides, we looked for presence of the same sequences in more distant phyla. A motif elicitation analysis (see Methods) revealed three separate sequence motifs in the *RPLP1* CNE, and three in the *RPLP2* CNE. These motifs are present in many different phyla (Fig. 5), including both deuterostomes and protostomes, making these the third and fourth known examples of bilaterian conserved regulatory elements (Bicores) [13]. These two conserved sequences were both early innovations in Animalia. The RPLP1 CNE was found in the genomes of the placozoan *Trichoplax adhaerens* and the cnidarian *Nematostella vectensis* (starlet sea anemone). *N. vectensis* also contains the RPLP2 CNE, as do the ctenophore *Mnemiopsis leidyi* (warty comb jelly) and the poriferan *Amphimedon queenslandica* (a demosponge). Both CNEs were present in the majority of species that we analyzed (*RPLP1*: 33/38 species analyzed, *RPLP2*: 23/38). Previously, the most ancient CNEs identified were found conserved between Deuterostomia and Cnidaria [11], thus dating back over 670 million years [12]. The CNE on *RPLP2* that we report here appears to have originated even earlier, being found in the Porifera. This CNE is thus likely over 700 million years old [8]. The CNE on *RPLP1* may also be older than 670 million years, depending on how the deep splits in the phylogeny of animals are eventually resolved [18].

**Fig. 6:**
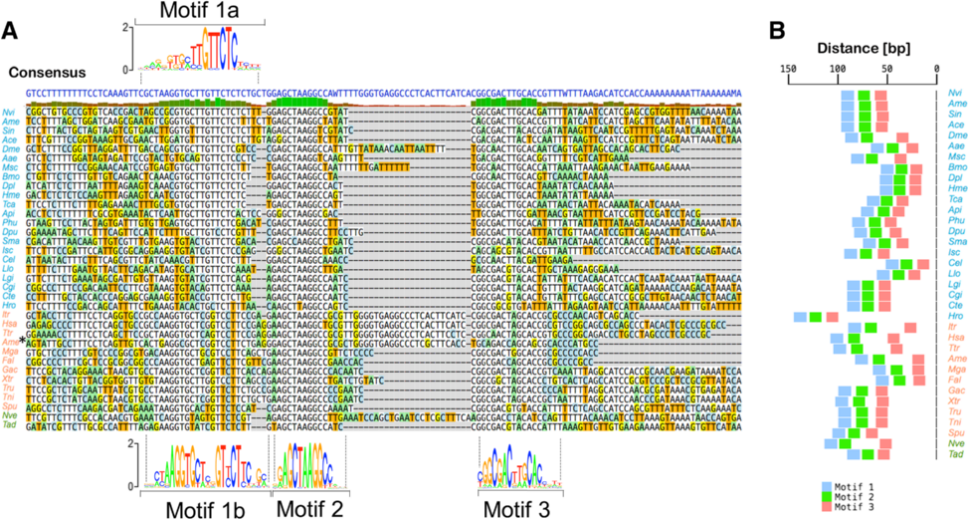
Evolution of RPLP1 CNE over at least 670 million years. **a** Alignment of RPLP1 CNE in all organisms where detected. Sequence logo diagrams of each conserved motif are shown below the alignment. Motif 1a is protostome-specific whereas motif 1b appears to be the ancestral and deuterostome form. Motifs 2 and 3 are variably spaced and are present in all phyla examined. Species name color scheme matches that of Fig. 5. **b** Diagram showing the position and spacing of each motif in each organism in relation to the translation start site. Genus/species abbreviations are defined in Additional file 2: Table S10

The conserved regions paint an interesting evolutionary story. Firstly, in the *RPLP1* CNE (Fig. 6), there are three distinct conserved sequences. The first conserved sequence appears to have two distinct forms; one found in protostomes (motif 1a) and another in deuterostomes (motif 1b), which appears to be the ancestral form as it is found in Cnidaria and Placozoa. The second and third motifs are found well conserved across both deuterostomes and protostomes, and are variably spaced; for example all mammalian species analyzed share a similar insertion between these two motifs. The relative position of the CNE is found conserved across all phyla (Fig. 6b), remaining within 150 bp of the translation start site.

**Fig. 7:**
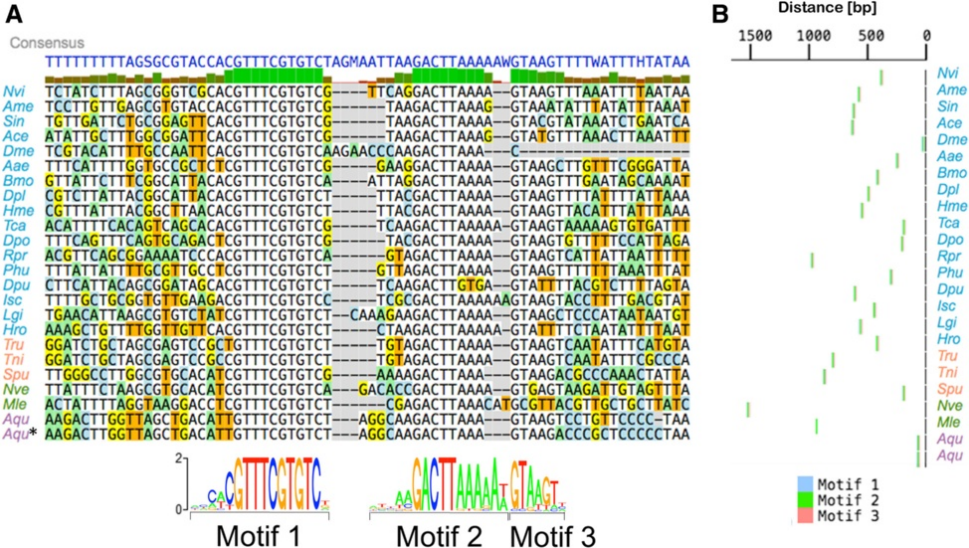
Evolution of RPLP2 CNE over at least 700 million years. a Alignment of RPLP2 CNE in all organisms where detected. The three distinct sequence motifs are shown aligned below the main alignment. Species name color scheme matches that of Figure 5. **b** Diagram showing the position and spacing of each motif in each organism in relation to the translation start site. Genus/species abbreviations are defined in Additional file 2: Table S10

The *RPLP2* CNE (Fig. 7) also appears to be described best as three distinct motifs. Motif 1 is exceptionally well conserved, with no variation at all across 10 bp. Motif 2 comprises a conserved region, generally followed by a short stretch of adenine nucleotides. Motif 3 is short and does not appear to be present in either *D. melanogaster* or *Mnemiopsis leidyi*. These observations make clear that these CNEs are functionally complex, being comprised of several discrete elements punctuated by less evolutionarily constrained sequence. This is in contrast to other kinds of conservation such as ultraconserved regions, where long stretches of nucleotides (>200 bp) are found perfectly conserved between human, rat, and mouse,[19] which can be in some cases deleted without a clear critical loss of function [20]. As a whole, the complexity, shared associated gene function, and age of these CNEs marks them as interesting targets for future study.

## Discussion

In this paper, we used a high stringency statistical approach to identify and characterize 322 ancient non-coding elements (Additional file 2: Table S4) which have remained conserved over large evolutionary distances. The bulk of the conserved sequences that we identified are specific to Hymenoptera, but nevertheless have been conserved in place for at least 180 million years of insect evolution (which occurs at a faster pace than vertebrate evolution [14]). A small proportion of the CNEs (20) that we identified were at least 350 million years old, with three stretching back further still. Two CNEs are found conserved in a range of the most basal animal clades across a wide variety of both vertebrates and invertebrates and are likely over 670 [12] and 700 million years old [8], likely the oldest CNEs described to date.

These two ancient CNEs are located in the 5’ UTRs of two genes that are known to interact with one another; *RPLP1* and *RPLP2*. The two protein products of these ubiquitously expressed genes, P1 and P2, form a heterodimer; two copies of which bind to the 60s acidic ribosomal protein P0 (coded by the gene *RPLP0*) to form the ribosomal stalk. The ribosomal stalk is involved in translational fine tuning and is crucial for the correct folding of many proteins [21]. The depth and breadth of conservation of these sequences is indicative of a fundamental regulatory role. Indeed, the 5’ UTR of *RPLP2* has already been shown to have a regulatory role in *Drosophila* [22], being sufficient to confer full translational control unto *RPLP2* as a non-translated gene in the early embryo, but not previously known to be conserved among animals. The fact that this CNE has been previously studied and identified as a regulatory element helps to validate the idea that other CNEs that we have identified are also functional regulatory elements. In *Drosophila*, the CNE we identified essentially spans the entirety of the *RPLP2* 5’ UTR, whereas in other organisms it is only a constituent part. In this study, we have characterized the motifs likely to be important for the function of this regulatory element and examined their evolution over time.

Most of the conserved regions identified in our analysis were found to be located within gene bodies as opposed to intergenic space, providing potential insights into a poorly understood class of regulatory elements. Our analysis revealed conserved secondary structures in the 5’ UTRs of several genes, examples including hairpin loops upstream of the *Ferritin* gene (an IRE), the *Paramyosin* gene, and the vital [23] regulatory [24] gene *Not1*. Secondary RNA structures such as hairpins can have important regulatory consequences, having the capacity to both enhance and inhibit translation. The effect of a hairpin on translation differs depending on the stability of the hairpin, its distance from the mRNA cap, and GC content [25]. These three hairpins have different fundamental characteristics, and thus likely perform their putative regulatory functions in different ways. The strong (−52.60 kcal/mol), and GC-rich (66 %) *Paramyosin* hairpin is likely able to present a significant barrier to translation at any distance from the cap, whereas the potential function of the weaker (~ − 15.00 kcal/mol) *Not1* hairpin is less obvious. The Not1 hairpin exhibits a complex conservation pattern, with near-perfect stem complementarity, tight positional conservation, and an associated conserved upstream AUG (uAUG), itself a tightly-suppressed [26] class of regulatory element.

The CNEs that we identified, confirming similar observations in other organisms [27], are associated with regulatory and developmental genes. This observation is consistent with the idea of regulatory gene cascades, that genes involved in regulation are themselves tightly regulated, allowing master regulators to exert overall control of gene regulation ‘programs’ to reprogram cells [28]. This result is augmented by the more specific observation that the deeply conserved set of 20 CNEs (> = 350 Myr), which are likely to be post-transcriptional regulatory elements, are associated with genes themselves involved in post-transcriptional regulation.

We also identified fundamental differences in basic sequence properties of the CNEs when compared with control sequences. GC content in CNEs is generally elevated (Additional file 1: Figure S8); sharply peaking on the CNE itself but also raised in the flanking regions. This result is informative as GC content is known to be important for regulation; it is associated with regulatory mechanisms such as nucleosome occupancy [29], aspects of secondary structure stability and effects on translation, and for example in chicken, variance in GC content in the 5’ UTR of genes can perhaps explain 10 % of the variation in expression level [30].

One important feature of many of the CNEs that we discovered is that their positions relative to the translation start site are conserved, i.e. that the position of the CNE is conserved as well as its sequence (Fig. 3b). When more reliable transcription start site data are available, it will be possible to examine whether putative transcriptional regulatory mechanisms that we identified are conserved relative to the transcription start site, or whether some of the translational regulatory mechanisms that we identified are more closely associated with the mRNA cap position than the translation start site. This positional information could be useful in detecting CNEs over large evolutionary distances, under the assumption that evolution sometimes proceeds by modifying the sequences of existing cis-regulatory CNEs without significantly changing their relative position [31].

## Conclusions

Overall, we have identified a large number of conserved sequence elements that, due to the strict false discovery controls that we have applied, and to previous experimental validation of a subset of these regions, are likely to be functionally important. It is our hope that each and every one of these regions will make interesting candidates for experimental analysis, helping to increase our understanding of regulation of gene expression, and particularly our understanding of regulatory elements in RNA.

### Availability of supporting data

We have made all of these regions along with our analysis of each freely available to browse on our interactive website http://waspatlas.com/cns_temp. The website is easy to browse, and includes details of the associated gene as well as detailed graphical representations of a number of CNE features, including sequence alignments, secondary structures, and positional information. Genomes of Nasonia vitripennis, Apis mellifera, Atta cephalotes, Solenopsis invicta, Drosophila melanogaster, Megaselia scalaris, Aedes aegypti, Bombyx mori, Danaus plexippus, Heliconius melpomene, Dendroctonus ponderosae, Tribolium castaneum, and Acyrthosiphon pisum were obtained from the core databases in Ensembl metazoa release 21 [32].

## Methods

### Detecting non-coding sequence conservation

All insect genomes were obtained from the core databases in Ensembl metazoa release 21 [32]. To search for conserved non-coding sequences, we extracted the 2 kb sequence upstream of each gene’s translation start site in *N. vitripennis* and compared this sequence with the sequence upstream of the orthologous gene in each comparator organism (Additional file 1: Figure S9). Our choice of 2 kb as an appropriate sequence length to analyse was twofold; firstly, based on the OGS v1.2 gene annotation, 2 kb is enough to cover over 95 % of 5’ UTR sequence, secondly, it is a computationally tractable amount of sequence given our resource and time constraints. Orthologs of *N. vitripennis* genes were computed using a pairwise reciprocal best BLAST hit (RBH) approach [33] based on the protein sequences (protein sequences from Ensembl metazoa release 21) of all transcripts in each genome. RBHs are calculated by a two-way comparison; for example if the best BLAST hit for a gene A in Nasonia is gene X in Drosophila, then it is called an RBH if and only if the best BLAST hit for gene X in Drosophila is gene A in Nasonia. The stringency of this criterion provides high-confidence orthologs. The number of *N. vitripennis* orthologs calculated for each genome is shown in Table S6. For our purposes of conducting a computationally intensive search for deeply conserved regions, this method was judged preferable to other, more comprehensive ortholog search methods such as orthoMCL [34] through a performance comparison of *Apis mellifera* and *Nasonia vitripennis* ortholog detection. While orthoMCL detects orthologs for more genes than the RBH method, RBH detects orthologs for most of these genes (>70 %, Additional file 1: Figure S11). Due to the higher sensitivity of orthoMCL to events such as gene duplication, the pairwise comparisons necessitated by an orthoMCL search (217,485) compared to those (best BLAST hits only) necessitated by an RBH search (7,435) would have been computationally prohibitive.

The 2 kb sequences were then extracted upstream of each translation start site. The choice of 2 kb allows us to capture both 5’ UTR and putative promoter sequence, while still being computationally tractable. If the 5’ end of a 2 kb sequence overlapped with a nearby gene, the sequence was truncated. If the sequence was entirely contained within another gene, then it was removed from the analysis entirely. Nasonia sequences were compared against sequences from each other species. The seaweeds algorithm provides optimal alignment scores for all pairs of possible windows across the two sequences, i.e. about 4 million short optimal alignments. The window length was chosen to be 50 bp. The alignment score was set to 1 for a match, 0 for a mismatch, and −0.5 for any gap. The rationale for this scoring matrix, and for using alignments in general, was to perform a search without *a priori* knowledge of the regions in question or the types of regulatory elements likely to be found. The choice of 50 bp is a variation on a previous study [35] which allows for greater sensitivity while maintaining specificity. An advantage of the window-based seaweeds algorithm [15] over other algorithms such as Smith-Waterman [36] is the avoidance of the “shadow effect” [37] where longer, but biologically less significant alignments may be computed while different, shorter alignments are ignored. Instead all windows are considered equally and results can be easily compared and tested for statistical significance as all sequences are of equal length.

To calculate a conservation score for a pairwise comparison, we take into account sequence conservation and punitively apply information about annotated repetitive elements to produce intermediate scores. These intermediate scores are then scaled from 0 to 1 using an lower (*L*) and upper (*U*) threshold; where scores below *L* are assigned a conservation score of 0, scores above *U* are assigned a conservation score of 1, and scores in between are defined on a sigmoid curve to reflect an initially exponential increase in confidence as scores increase above the lower threshold, which levels off as scores approach the upper threshold reflecting saturating confidence. The results of all the pairwise alignments were bundled together to form one inclusive dataset. Essentially, this step involves identifying individual small CNEs which actually map to the same sequence, and combining them into a single CNE (illustrated in Additional file 1: Figure S10). During the bundling process, significantly overlapping alignments, i.e. individual CNEs which score above the lower bound but which are not disjoint from one another, were merged together into longer regions and significant regions in two or more species which mapped to the same subsequence in *N. vitripennis* were identified and merged into a single CNE. Each potential merged CNE is then assigned a *combined conservation score* (CCS); the combination of the conservation scores from each pairwise comparison. The CCS is computed using the following formula:

1 $$ 1\ \hbox{-}\ {\Pi}_i\ \left(1\ \hbox{-}\ {P}_i\right) $$

Where *P* is the maximum conservation score for a potential CNE in a species, and i indexes the species.

To set the appropriate parameters for detecting conservation, we aligned randomly paired upstream sequences (*pseudo-orthologs*) to get an idea of what scores could be expected by chance. Using real non-orthologous genomic sequence as a control is preferable to using randomized or ‘shuffled’ sequence as it maintains the complex sequence makeup of true genomic sequences, whereas in shuffled sequences these motifs will be depleted providing a less stringent control. We first identified candidate CNEs by running the pseudo ortholog sequences and the true ortholog sequences with lower *L* and *U* parameters (*L*: 80 *U*: 94), and setting a CCS threshold at the level where no conservation was detected in the pseudo ortholog set. The lower and upper bound parameters were initially set based on examination of pairwise comparison histograms; the lower threshold was set at the point where scores were unlikely to be meaningful (i.e. where the control set shows a similar number of CNEs), and the upper threshold at a level where no sequences were reported in the control, as performed by Baxter *et al*. [35]. These candidate CNEs were filtered for similarity to known coding sequences, and after increasing the parameters to the level where no conservation was detected in the pseudo-ortholog control (*L*: 87 *U*: 100), we scored these candidate CNEs for conservation producing a final filtered set of 322 CNEs. At this point of filtering, the lower and upper bound are simply used to define a continuum of confidence scores for CNEs already known to be significant.

### Filtering for coding sequences and pseudogenes

To ensure that the CNEs were unlikely to contain unannotated coding sequences or pseudogenes, we utilized BLASTX 2.2.27+ [38] to filter out such sequences. To set an appropriate filtering threshold, we first randomly permuted the nucleotide sequences in the set to be filtered. These sequences were then scored against the nr protein database [39] using BLASTX, and the minimum (most significant) E-value score was noted as the most significant hit likely to be produced by random sequences with identical nucleotide composition to the set to be filtered. Once this threshold was set, the true sequences were scored against nr using BLASTX, and any sequence scoring below this threshold was discarded. The thresholds and number of sequences filtered by this method can be seen in Additional file 2: Table S7.

### Pseudo CNE comparisons

To elucidate properties of the CNEs through comparison, we generated a set of *pseudo CNEs*. The set of 359 pseudo CNEs that we obtained were created by aligning pseudo orthologs with relatively low threshold parameters (*L*: 80 *U*: 94), applying a CCS cutoff of 0.528 to retrieve a similar number of sequences to the true CNEs, and performing BLASTX filtering. Pseudo CNEs constitute a good control as they are similar to true CNEs in every way but for the fact that they do not represent true orthologous sequences. By comparing the CNEs with these pseudo CNEs, we can therefore identify properties likely to be characteristic of the truly conserved non-coding sequences.

All comparisons were performed on the *N. vitripennis* versions of each CNE. GO term overrepresentation analysis was performed on both the CNEs and pseudo CNEs using BiNGO [40], a plugin for Cytoscape [41]. We used annotation obtained from the Gene Ontology Consortium [42] (data-version: 2013-08-23) and tested against a background annotation set, formed by combining annotation derived from the *D. melanogaster* Ensembl homologs of the full set of *N. vitripennis* genes, ensembl GO annotation, and the official gene set 2 GO annotation on NasoniaBase [43, 44]. Statistical comparisons of the distributions of GC content, CpG observed/expected, CNE length, and CNE position were performed using the Wilcoxon rank sum test in R [45]. *P*-values of 2.2e-16 are at the floating point precision limit (p ~ 0 at machine precision). For each CNE, the distance from translation start site was calculated as the distance from the 3’ end of the CNE from the 5’-most annotated translation start site of each gene. Unless indicated otherwise, all comparisons are between the *N. vitripennis* true CNEs and the *N. vitripennis* pseudo CNEs.

### Nucleosome occupancy prediction and GC content analysis

We used the nucleosome occupancy prediction software described in [46] to predict nucleosome occupancy within each CNE and in the flanking region. We extracted the sequence of each CNE along with flanking sequence in order to obtain a 10 kb sequence centered on each CNE. Sequences containing Ns were removed as per the software requirements. This step removed a significant proportion of CNEs - 142 of 322 CNEs (44 %). Control sets were produced by extracting, for each CNE, a region with the same properties (length, and distance from translation start site) upstream of a randomly selected gene. 10 random sets were created, and the results from these controls were averaged and the standard deviations calculated. For the GC content comparison, sequences were extracted in the same way and GC content was measured in a sliding window (50 bp window size, 10 bp step size) along each sequence using a custom Perl script.

### 5’ UTR analysis

To get an estimate of how many sequences were conserved in transcribed regions as opposed to non-transcribed regions, we split each CNE into all possible 20-mers and used bowtie2 v2.0.5 [47] to map each sequence to the *N. vitripennis* evidential gene transcriptome dataset [43, 44] supplemented with RNA-seq data (data available on http://www.waspatlas.com), as well as the Ensembl version of the official gene set OGS v1.2 augmented with the same data. A 20-mer was counted as transcribed if it mapped to either transcriptome, and the percentage of mapped to unmapped reads was calculated to give an overlap percentage for each CNE.

To test for the presence of conserved secondary structures, the SCI (structure conservation index) was calculated for each CNE alignment and used to compute the probability of a conserved secondary structure. The SCI is defined as the minimum free energy of the consensus folding structure divided by the mean minimum free energy (MFE) of each sequence in the alignment folded independently [48]. Sequences in alignments with high structural conservation will show similar energies whether allowed to fold independently or forced into the consensus structure. A high SCI (close to 1) therefore indicates a well-conserved structure, and an SCI of more than 1 may indicate the presence of compensatory mutations. We implemented the SCI in Perl using RNAfold [49] and RNAalifold [50], and used code from [51] to implement a shuffling procedure as a control. Alignments are shuffled on a column-by-column basis, keeping the overall conservation pattern intact. By generating sets of shuffled alignments in this way, we can thus calculate the probability that the conservation of RNA secondary structure in the true alignment is exceptionally strong. For each CNE, we generated 1000 control sequences, calculated the Z score distribution, and used this to generate an empirical *p*-value. 29 CNEs showed conserved structure at *p* < 0.05, and 11 at *p* < 0.01.

### Motif overrepresentation

To test for overrepresentation of motifs, we used a set of 1038 position weight matrices, including matrices from JASPAR [52] and PLACE [53] and followed the procedure in [35]. We reduced redundancy by performing hierarchical clustering based on the Hellinger distances between matrices, yielding a set of 735 representative matrices at a threshold of 1.5. The matrix with the median entropy was selected to be the representative of each cluster. Motifs were tested for overrepresentation using a binomial test taking into account the strength of the matches. We produced 100 control sets using the same method as in the nucleosome occupancy prediction/GC content analysis section, with the exception that if we found Ns in the true CNEs then Ns were inserted into each control CNE in the same positions. A matrix was counted as over-represented if the binomial p-value in the true CNE set was lower than the p-value in all control sets, and underrepresented if it was higher than the p-value in all control sets. Of the 735 representative matrices, 88 were found to be under-represented compared to the controls, and 35 were over-represented. As with the nucleosome occupancy analysis, this analysis was highly affected by GC content; in general, matrices with high GC content were found to be overrepresented whilst those with low GC content were found to be underrepresented. GC content of a matrix was measured proportionally with the weight of each position; a matrix with several highly weighted G or C nucleotides will thus have higher GC content than a matrix with G or C nucleotides that have low weight.

### Not1 CNE characterization

To test for presence or absence of the Not1 CNE, the consensus sequence from the original CNE alignment was scored against the 100 bp upstream sequences of Not1 homologs in all organisms available in Ensembl metazoa using BLAST [38]. The E-value distribution was plotted, and sequences with E-values lying outside of the main distribution (E < 0.001) (Additional file 2: Table S8) were aligned, and the 30 bp hairpin loop sequences extracted. The stabilities of the hairpins were predicted using RNAfold [49] and sequence logo diagrams prepared using the seqLogo [54] package in R.

### Motif elicitation and RPLP1/RPLP2 analysis

MEME [55] was used to elicit motifs from the *RPLP1* and *RPLP2* CNEs. After initial exploratory analysis, we searched for the 3 best motifs in the sequences of both CNEs, looking for motifs from 6 to 13 bp in the *RPLP1* CNE, and from 6 to 15 bp in the *RPLP2* CNE. For the *RPLP1* CNE, 500 bp sequences upstream of all RPLP1 homologs analyzed were used, and 2 kb sequences for the *RPLP2* homologs analyzed. Due to total sequence length restrictions, the *RPLP2* motif elicitation analysis was first done with a seed elicitation followed by analysis of the remaining sequences. 3 motifs were extracted for the *RPLP1* CNE, and 2 for the *RPLP2* CNE, one of which was manually split into two upon further inspection. Sequences were inspected for presence or absence of the CNE motif components, and sequences containing the CNEs were aligned based on the motif positions. Distances of the motifs from translation start sites were calculated and plotted. Sequence logo diagrams were prepared using the SeqLogo [54] package in R. All genes and organisms used in this analysis can be found in Additional file 2: Table S9.

The motif elicitation analysis was repeated using a phylogeny-aware method, Phylogibbs [56]. The motif-based alignments produced by MAST [55] were cut to 17 sequences due to memory constraints, and used as inputs for the algorithm. Phylogenetic trees were constructed using divergence estimates from timetree [8], and blanket proximity values were assigned to branches based on the approximate figure of 0.85 proximity given in the Phylogibbs documentation for the mouse-rat divergence time of 22.6 Myr. The analysis for the *RPLP1* CNE revealed three significant motifs overlapping the motifs produced by MEME. Similar results were also obtained for the *RPLP2* CNE. The full results of this analysis including the elicited PSSMs can be found in Additional files 3 and 4.

## Additional files

Additional file 1: Figure S1. Association of CNEs with predicted nucleosome occupancy. **Figure S2.** Relationship of transcription factor binding site motifs with GC content. **Figure S3.** CNE sequences show an underrepresentation of upstream ATG trinucleotides. **Figure S4.** *Ferritin* 5’ UTR iron response element (IRE) alignment diagram. **Figure S5.** Conserved CNE is a novel hairpin in the 5’ UTR of the *Paramyosin* gene. **Figure S6.** Uncharacterized Osiris gene cluster contains several CNEs. Conserved upstream sequence location relative to the coding sequences and combined conservation scores of six genes in the *Osiris* cluster. **Figure S7.** Three CNEs in the 5’ UTR of *UNR*. Conserved upstream sequences located in the 5’ UTR of the master regulator *UNR*. **Figure S8.** Association of CNEs with GC content. Average GC content across the genomic regions containing the CNEs, compared to control sets. **Figure S9.** A schematic diagram of the alignment strategy used in this study. **Figure S10.** A schematic diagram of the bundling strategy. Three pairwise CNEs found conserved between *N. vitripennis* and two other species are bundled into a single CNE. **Figure S11.** Comparison of ortholog identification methods. (PDF 340 kb) Additional file 2:
**Supplementary tables Table S1.** Full list of overrepresented GO terms (q < 0.01) for the set of 322 true CNEs (hypergeometric test). **Table S2.** Full list of overrepresented GO terms (q < 0.01) for the set of 359 pseudo CNEs (hypergeometric test). **Table S3.** Full list of overrepresented GO terms (q < 0.01) for the set of 20 ancient CNEs (hypergeometric test). **Table S4.** Full list of 322 detected CNEs. **Table S5.** Homeobox-domain containing genes with detected CNEs. **Table S6.** RBH orthologs found by species analysed. **Table S7.** BLASTX filtering of CNE sets. **Table S8.** Significant (E < 0.001) results of a BLASTN search for the onsensus Not1 CNE in 100 bp upstream regions of the Not1 translation start site. **Table S9.** Genes and organisms used in the *Not1* CNE analysis. **Table S10.** Abbreviations used in Figure 6 and Figure 7. (XLSX 73 kb) Additional file 3:
**Standard Phylogibbs output for the** ***RPLP1*** **CNE showing elicited motifs, their sequences, the organisms used and their approximate phylogenetic relationships to one another.** (TXT 7 kb) Additional file 4:
**Standard Phylogibbs output for the** ***RPLP2*** **CNE showing elicited motifs, their sequences, the organisms used and their approximate phylogenetic relationships to one another.** (TXT 8 kb)
