# Supplementary material for: Analysis of 5’ gene regions reveals extraordinary conservation of novel non-coding sequences in a wide range of animals
Source: BMC Evol Biol. 2015 Oct 19;15:227. doi: 10.1186/s12862-015-0499-6 (PMC4613772; doi:10.1186/s12862-015-0499-6)
Supplement: Additional file 1: Figure S1. — Association of CNEs with predicted nucleosome occupancy. Figure S2. Relationship of transcription factor binding site motifs with GC content. Figure S3. CNE sequences show an underrepresentation of upstream ATG trinucleotides. Figure S4. Ferritin 5’ UTR iron response element (IRE) alignment diagram. Figure S5. Conserved CNE is a novel hairpin in the 5’ UTR of the Paramyosin gene. Figure S6. Uncharacterized Osiris gene cluster contains several CNEs. Conserved upstream sequence location relative to the coding sequences and combined conservation scores of six genes in the Osiris cluster. Figure S7. Three CNEs in the 5’ UTR of UNR. Conserved upstream sequences located in the 5’ UTR of the master regulator UNR. Figure S8. Association of CNEs with GC content. Average GC content across the genomic regions containing the CNEs, compared to control sets. Figure S9. A schematic diagram of the alignment strategy used in this study. Figure S10. A schematic diagram of the bundling strategy. Three pairwise CNEs found conserved between N. vitripennis and two other species are bundled into a single CNE. Figure S11. Comparison of ortholog identification methods. (PDF 340 kb) [file 12862_2015_499_MOESM1_ESM.pdf]

Analysis of 5’ gene regions reveals extraordinary conservation of novel non-coding sequences in a wide range of animals

Nathaniel J. Davies, Peter Krusche, Eran Tauber and Sascha Ott

September 2015

Additional figures S1-S11

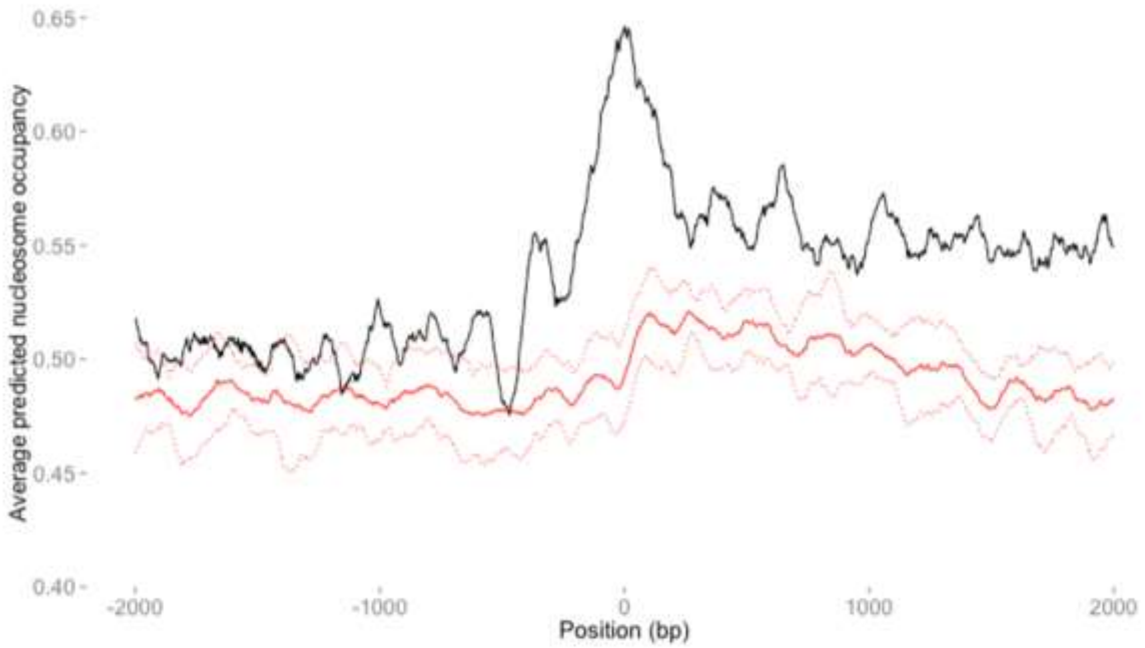

Figure S1. Association of CNEs with predicted nucleosome occupancy.
Average predicted nucleosome occupancy across the genomic regions containing the CNEs, compared to control sets. Predictions are shown for fragments of 4 kb including the CNEs in the center. Fragments are oriented such that the associated gene is 3’ of the CNE. For each control set “mock CNEs” upstream of randomly selected genes are matched to real CNEs in terms of distance to gene, size, and absence of known repeats. Averages of the control sets are plotted in red, with +/- standard deviations of 10 control sets as dotted lines. Nucleosome occupancy shows a significant peak in the center of CNEs.

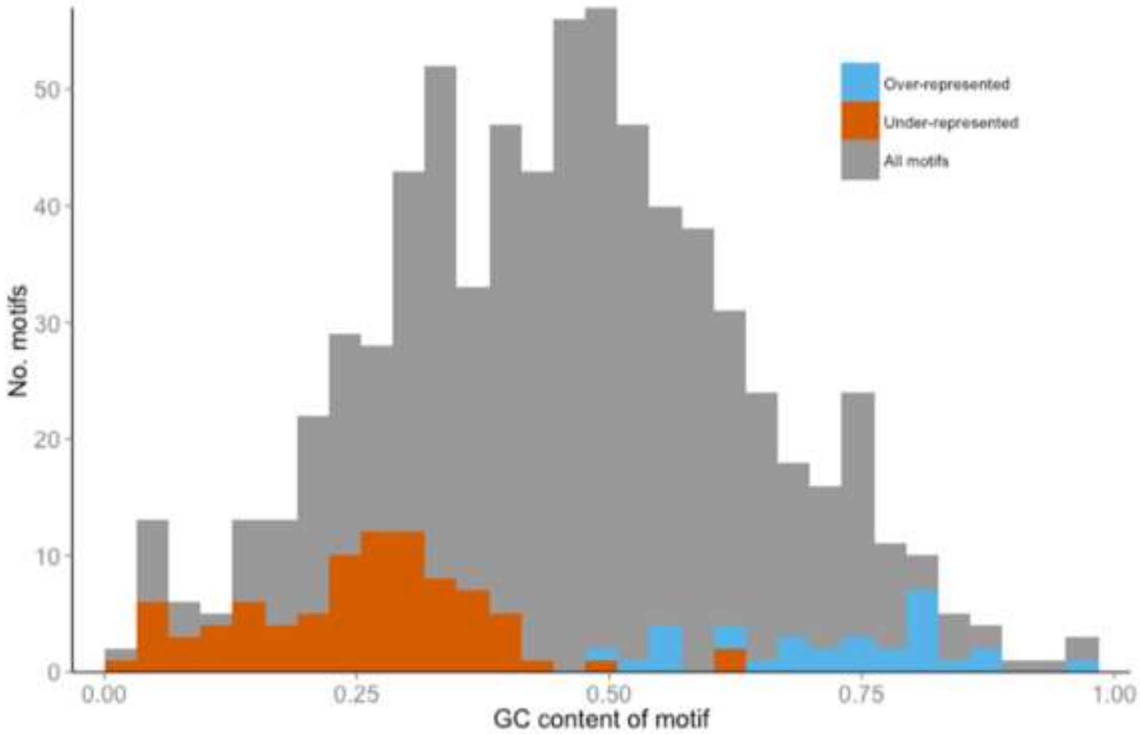

Figure S2. Relationship of transcription factor binding site motifs with GC content. Transcription factor binding site motifs plotted by motif GC content. Those motifs which were underrepresented in the CNEs compared to the control are plotted in red, and those overrepresented are shown in blue. A clear disparity in GC content is visible between the under and over-represented motifs.

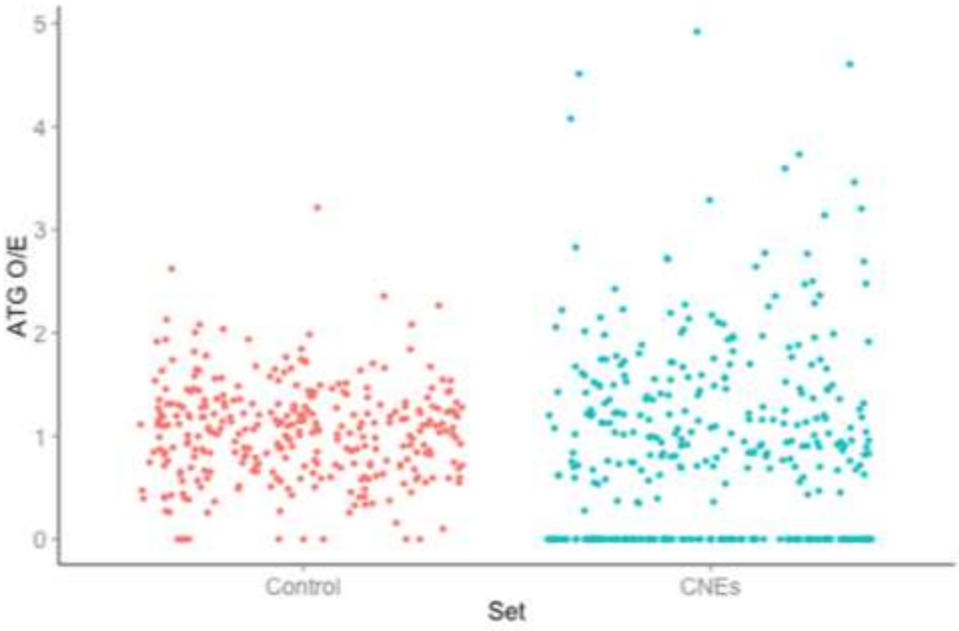

Figure S3. CNE sequences show an underrepresentation of upstream ATG trinucleotides. Computing the ATG observed/expected ratio reveals an overrepresentation of ATG-free CNEs ($p<0.01$, Wilcoxon sum rank test), but similar usage otherwise.

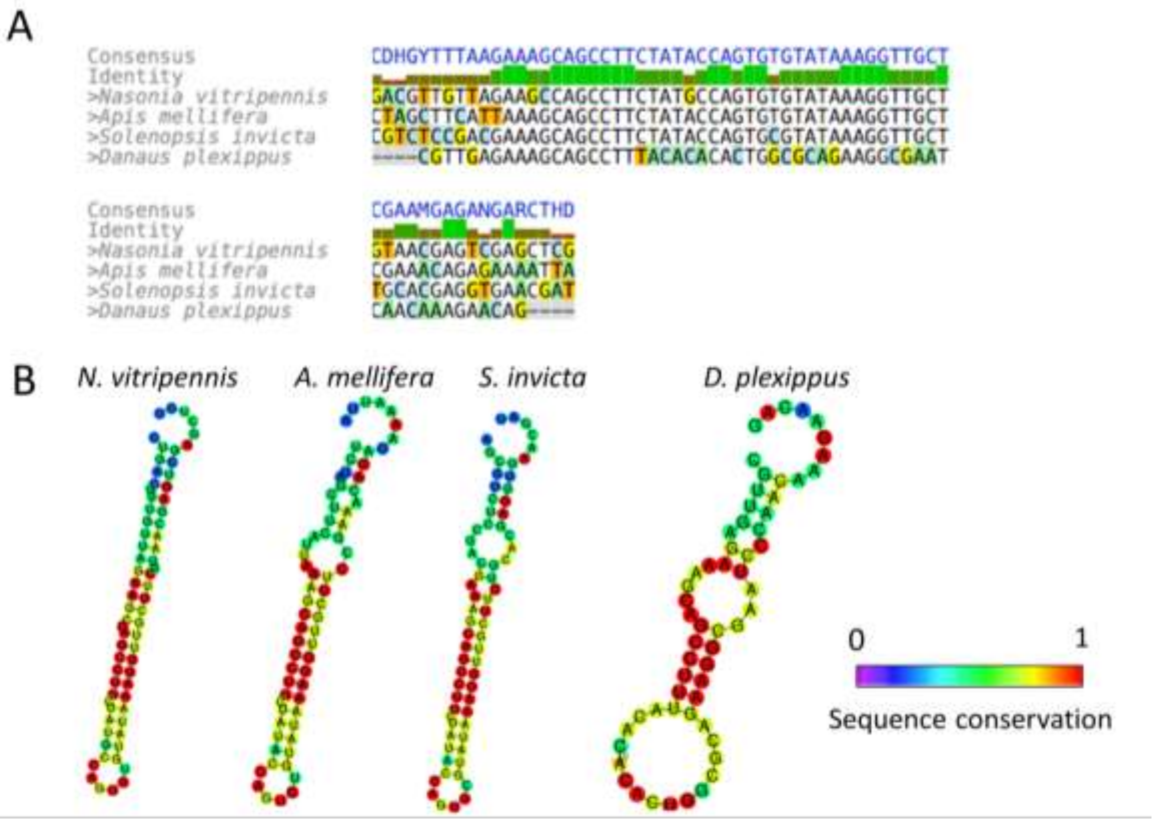

Figure S4. Ferritin 5’ UTR iron response element (IRE) alignment diagram. (A) predicted secondary structures (B) colored by sequence conservation based on the alignment. Sequence (A) and loop (B) are clearly altered in *D. plexippus*, but otherwise almost perfectly conserved.

Figure S5

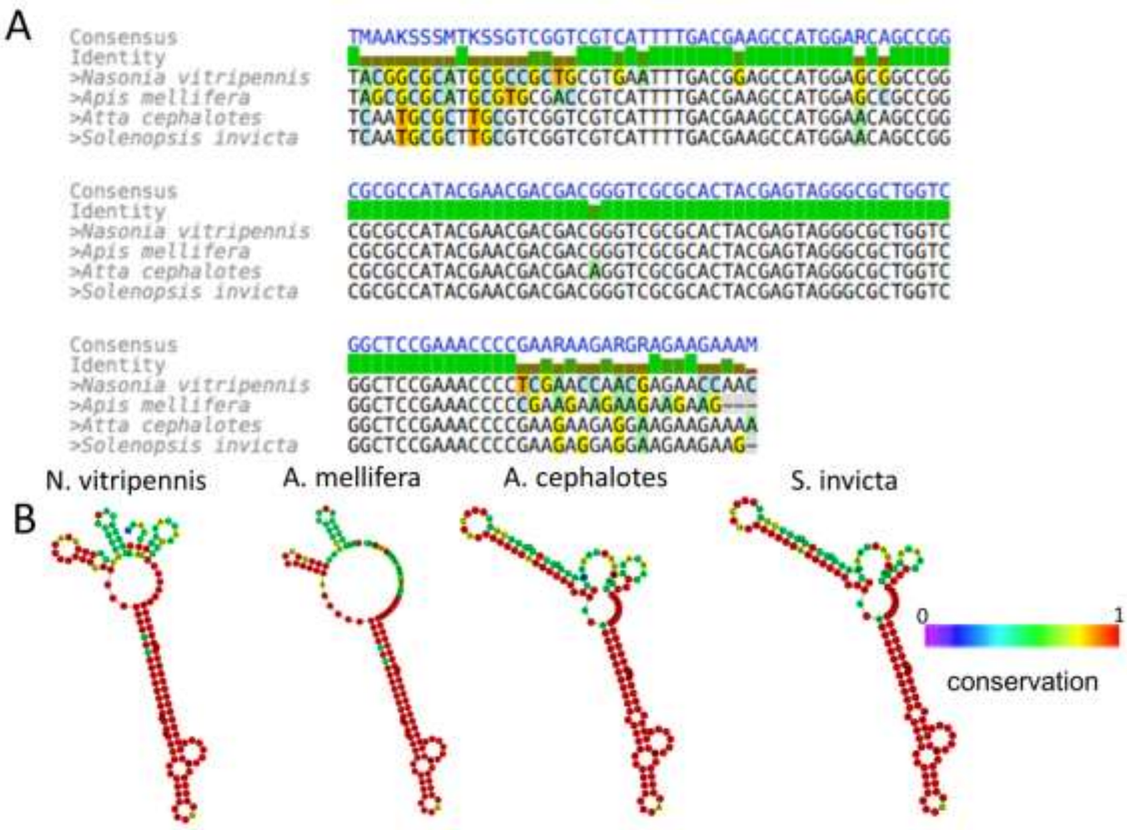

Figure S5. Conserved CNE is a novel hairpin in the 5’ UTR of the *Paramyosin* gene. Conserved *Paramyosin* hairpin, sequence alignment (A), and predicted secondary structures (B) colored by sequence conservation based on the alignment.

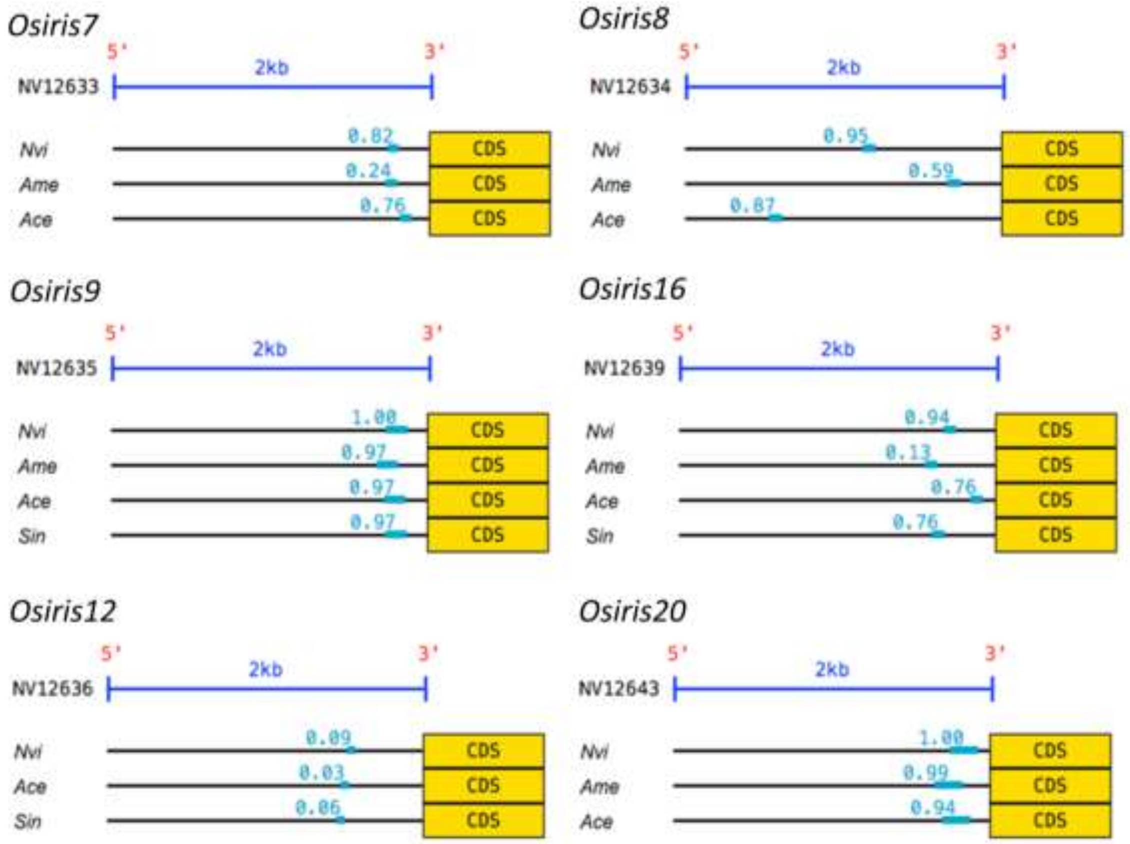

Figure S6. Uncharacterized Osiris gene cluster contains several CNEs.
Conserved upstream sequence location relative to the coding sequences and combined conservation scores of six genes in the Osiris cluster.

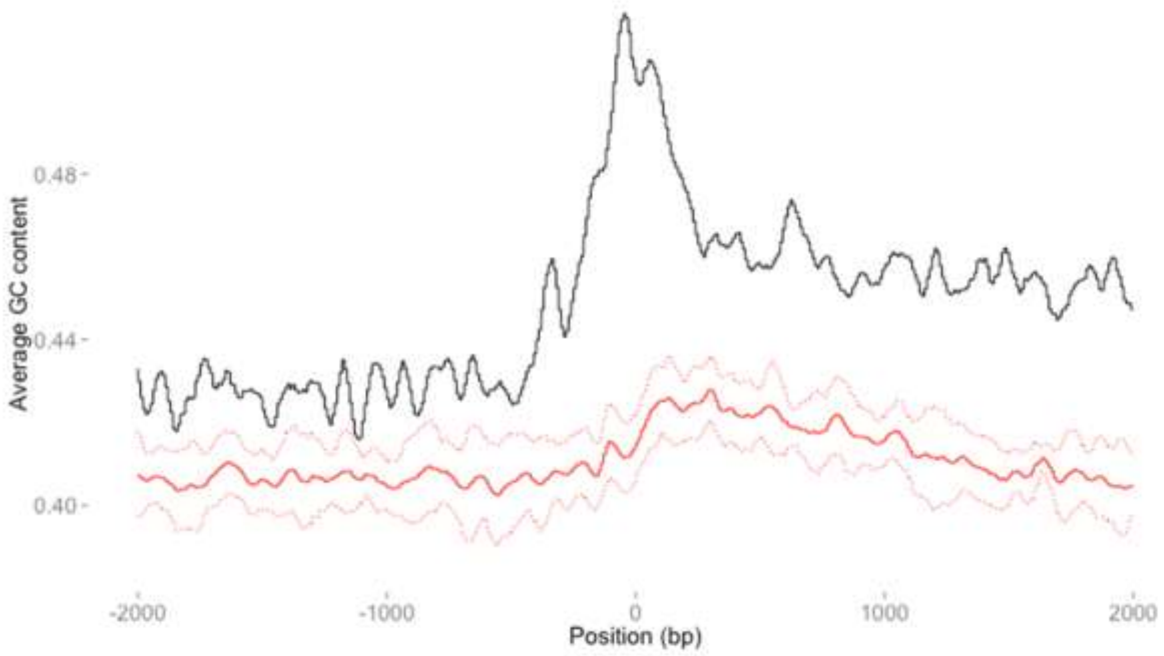

Figure S7. Association of CNEs with GC content. Average GC content across the genomic regions containing the CNEs, compared to control sets. Data is shown for fragments of 4 kb including the CNEs in the center. Fragments are oriented such that the associated gene is 3' of the CNE. For each control set "mock CNEs" upstream of randomly selected genes are matched to real CNEs in terms of distance to gene, size, and absence of known repeats. Averages of the control sets are plotted in red, with +/- standard deviations of 10 control sets as dotted lines. A clear GC content peak is visible at the center of the CNEs.

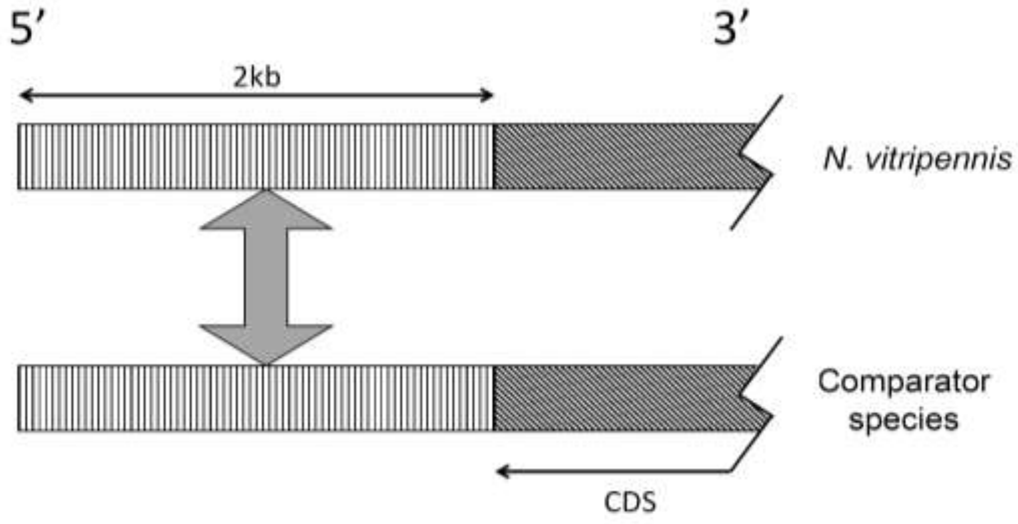

Figure S8. A schematic diagram of the alignment strategy used in this study. 2 kb regions are taken upstream of the coding sequence of a gene in *N. vitripennis* and its homolog in a comparator organism.

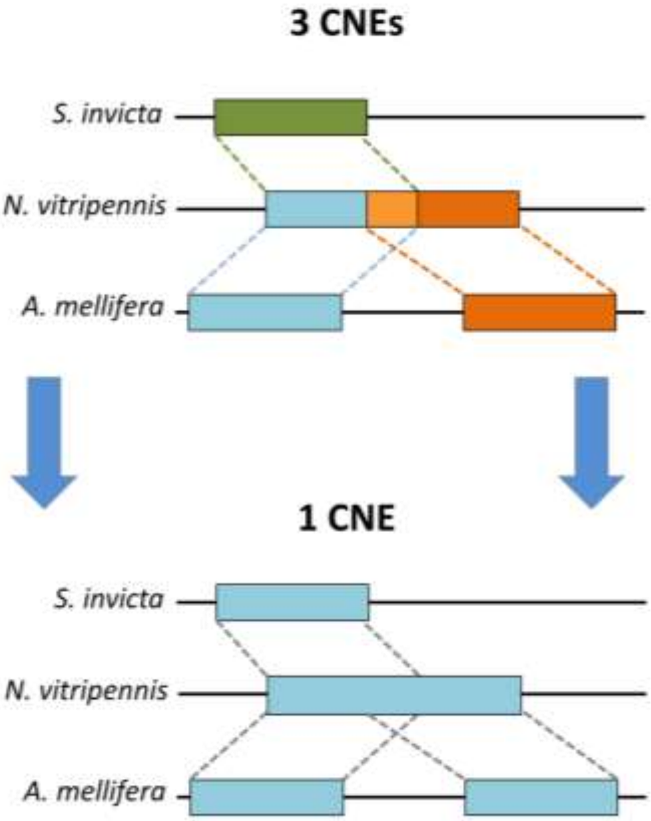

Figure S9. A schematic diagram of the bundling strategy. Three pairwise CNEs found conserved between *N. vitripennis* and two other species are bundled into a single CNE.

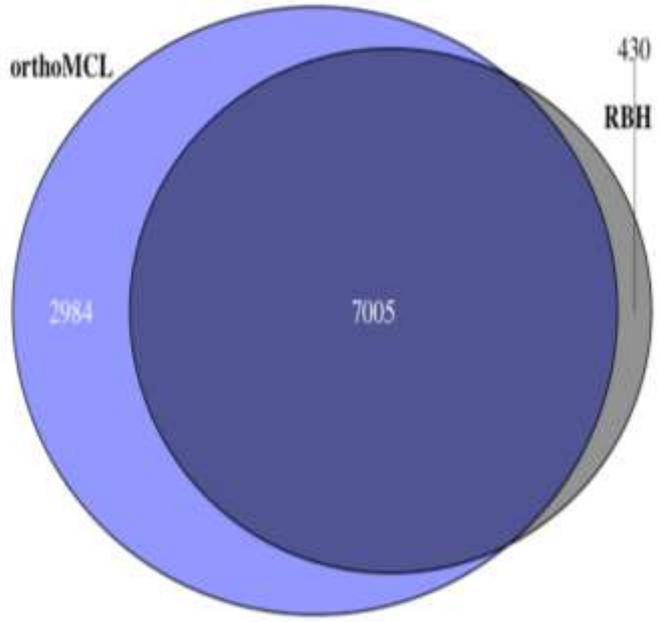

Figure S10. Comparison of ortholog identification methods. Venn diagram illustrating a comparison of the number of genes with detected orthologs by the RBH method (grey, right) with the number of genes with detected orthologs by the orthoMCL method (blue, left).
